# Psychological responses among nurses caring for patients with COVID-19: a comparative study in China

**DOI:** 10.1038/s41398-020-00993-1

**Published:** 2021-05-06

**Authors:** Honggang Ren, Xingguang Luo, Yincheng Wang, Xiaoyun Guo, Huiru Hou, Yong Zhang, Pengcheng Yang, Fang Zhu, Chao Hu, Runsheng Wang, Yu Sun, Yingzhen Du, Qin Yin, Guogang Xu, Hui Zuo, Qinyong Hu, Yahui Wang

**Affiliations:** 1 Department of Internal Medicine, Tongji Medical College, Huazhong University of Science and Technology, Wuhan, China; 2 Department of Psychiatry, Yale University School of Medicine, New Haven, CT USA; 3 Department of Psychology, Tsinghua University, Beijing, China; 4 Shanghai Mental Health Center, Shanghai Jiao Tong University School of Medicine, Shanghai, China; 5 The Second Medical Center & National Clinical Research Center for Geriatric Diseases, Chinese PLA General Hospital, Beijing, China; 6 Tianjin Mental Health Center, Tianjin, China; 7 Cancer Center, Renmin Hospital of Wuhan University, Wuhan, China; 8 Department of Anesthesia and Perioperative Medicine, Western University, London, Canada; 9 Department of Geriatric Medicine, The Eighth Medical Center of Chinese PLA General Hospital, Beijing, China; 10 Department of Respiratory Medicine, The Second Medical Center & National Clinical Research Center for Geriatric Diseases, Chinese PLA General Hospital, Medical College of PLA, Beijing, China; 11 Department of Otorhinolaryngology, Union Hospital, Tongji Medical College, Huazhong University of Science and Technology, Wuhan, China; 12 Department of Respiratory and Critical Care Medicine, Hubei provincial hospital of Integrated Chinese and Western Medicine, Wuhan, China; 13 School of Public Health, Medical College of Soochow University, Suzhou, China

**Keywords:** Depression, Human behaviour

## Abstract

Frontline healthcare nurses devoted themselves to deal with the outbreak of COVID-19, saving many lives. However, they are under incredible unknown psychological pressures with a considerable risk of infection. In this study, a self-administered questionnaire was used to survey 593 frontline nurses in Wuhan City and non-Hubei provinces for psychological responses from March 1 to March 10, 2020. Compared with nurses outside Hubei Province, those working in Wuhan were more likely to feel physically and mentally exhausted. Their probable depression and anxiety were significantly higher than those of nurses outside Hubei province (31.2%, 18.3% vs. 13.8%, 5.9%). Correspondingly, the depressive symptoms were more often reported in the Wuhan group (70.8% vs. 41.4%). Although Wuhan received wishes, concerns, and abundant psychological and material resources from all of the world, the survey-based study found that frontline nurses in Wuhan still had higher depression and anxiety with less social support compared with nurses from non-Hubei provinces. Unexpectedly, only 4.0% of nurses have sought psychological assistance. These findings suggested that the short-term psychological impact of frontline nurses in Wuhan during the COVID-19 outbreak was extremely high compared with nurses outside Hubei Province. This research enlightened the efficient integration of psychological resources, the optimization of the nurse emergency psychological assistance system, and the mental health care of medical staff during the outbreak of epidemics.

## Introduction

In December 2019, unnamed pneumonia appeared in Wuhan, China, with unexplained pathogenesis. WHO later named the disease as Coronavirus Disease (COVID-19), and the causative agent was identified as the coronavirus COVID-19^1^. During the first 2 months of the outbreak, COVID-19 spread rapidly throughout China, causing varying degrees of illness, and many parts of the world now are also under severe conditions^2,3^. As of March 1st, 26 Chinese healthcare workers had died of COVID-19. In particular, frontline nurses face high infection risks, including close contact and long working hours caring for COVID-19 patients, while the implementation of psychological intervention services encountered obstacles, as the medical staff was reluctant to participate in the group or individual psychological interventions provided to them^4^. Medical curative treatments were in clinical trials with therapeutic effects founded. However, frontline nurses are still under significant mental and working pressures, which lead to variable potential risks though considerable assistance from worldwide^5^. Therefore, the psychological responses to this pandemic crisis amongst nurses are of significant interest to both scientists and public health authorities.

The Wuhan city, Hubei province, is at the center of the pandemic in China. It has had the vast majority of cases and deaths compared to cities outside Hubei province in China^6^. This outbreak also may have led to psychological conflicts between the responsibility to care for the ill and their right to protect themselves from potential varying degrees of illness^7^. In light of a possible recurrence of an outbreak, it is critical to understand the nature of the psychological responses of healthcare frontline workers^8^. Therefore, we hypothesize that the psychological impact of COVID-19 on nurses in Wuhan was greater than that of their colleagues outside Hubei province. However, we hypothesize that they had higher social support because the worldwide provided abundant psychological assistance resources and supports for the frontline nurses in Wuhan. A better understanding of such impacts is important for developing methods and related service system of preventative interventions for the future mass health crisis.

Therefore, a multi-center comparative study in China was performed to assess the psychological responses among nurses dealing with the COVID-19 in Wuhan and outside Hubei province.

## Methods

### Study design and participants

This cross-sectional study is a multi-center comparative survey on the psychological condition of frontline healthcare nurses in Wuhan and non-Hubei cities in China during the COVID-19 pandemic. Data were randomly collected by a self-administered questionnaire with 177 items (taking around 20 min) via the WeChat-based survey program Questionnaire Star, between March 1 and March 10, 2020. The questionnaire incorporated information including demographic data, psychological scales, and psychological interventions received by the participants. A total of 593 participants responded, and all of them were involved in the study.

This study was approved by the Ethical Committee of the Union Hospital, Tongji Medical College, Huazhong University of Science and Technology. Signed informed consent was obtained online from all participants.

### Instruments

Probable current depression and depressive symptoms in the past 2 weeks were assessed using the Patient Health Questionnaire-922 (PHQ-9)^9^. PHQ-9 is a standardized 4-point scale with 9 items. Clinically, the answers to these questions are assigned a score from 0 to 3 (indicating “Not at all” to “Nearly every day”), for a total range of 0–27. This scoring system can help classify patients according to the degree of their reported symptoms. We considered PHQ-9 as a continuous depressive symptom score (range 0–27) and a binary indicator for depressive symptoms (PHQ-9 ≥ 5) and probable major depression (PHQ-9 ≥ 10). Similarly, the Generalized Anxiety Disorder 7-item (GAD-7) 4-point scale was used to identify anxiety cases^10^. Social Support Rating Scale (SSRS) was also used to measure the perception of social support^11^. It composed of 10 items, measuring three dimensions of social support: subjective support, objective support, and support-seeking behavior.

### Other measures

The demographic data included gender, age, educational level, professional qualifications, years of work, etc. Of note, we also collected data on psychological assistance sought (types and sources of assistance, and the use of sedative/hypnotic drugs, etc.).

### Statistical analysis

Categorical variables were summarized as counts and percentages and were compared using Chi-square tests between the groups. Continuous variables were described as mean and standard error and were compared using *t*-test analysis. Statistical analyses were performed using SAS (Version 9.4, The SAS Institute, Cary, NC). A two-sided *p*-value < 0.05 was considered statistically significant.

## Results

### Demographic characteristics of the participants

Of the 593 participants included in this study, 202 were in Wuhan, Hubei province. The other 391 were in other cities outside the province, including Beijing and Shanghai. The vast majority of the participants in Wuhan (96.5%) and outside the province (91.3%) were female. There was no significant difference in the age of the nurses or time dealing with the COVID-19, as shown in Table 1. Most of the participants came from top tertiary hospitals in the two groups (78.2% vs. 68.3%). The proportion of participants working in the intensive care unit (ICU) was slightly higher in the Wuhan group (4.5%) than that in the non-Hubei group (1.8%). Nurses in Wuhan were more likely to change their working places.

**Table 1:** Demographics and baseline characteristics of nurses on admission.

	Wuhan (%)/*N*	Non-Hubei (%)/*N*
Gender (female)	195 (96.53)	357 (91.3)
Age		
20–30	98 (48.51)	197 (50.38)
30–40	72 (35.64)	126 (32.23)
40–50	25 (12.38)	46 (11.76)
>50	7 (3.47)	22 (5.63)
Education		
College	78 (38.61)	116 (29.67)
Undergraduate	119 (58.91)	264 (67.52)
Master	5 (2.48)	11 (2.81)
Time participate the current work (week)		
1	4 (1.98)	52 (13.3)
2	34 (16.83)	31 (7.93)
3	20 (9.9)	24 (6.14)
4	23 (11.39)	85 (21.74)
5	23 (11.39)	66 (16.88)
6	23 (11.39)	37 (9.46)
7	21 (10.4)	10 (2.56)
8	9 (4.46)	13 (3.32)
>8	45 (22.28)	73 (18.67)
Professional qualifications		
Nurse practitioner	64 (31.68)	120 (30.69)
Nurse	72 (35.64)	110 (28.13)
Supervisor’s carer	54 (26.73)	132 (33.76)
Deputy Director’s nurse	10 (4.95)	25 (6.39)
Chief nurse	2 (0.99)	4 (1.02)
Years of work		
≤10 years	119 (58.91)	232 (59.34)
11–20	50 (24.75)	87 (22.25)
21–30	22 (10.89)	50 (12.79)
>30 years	11 (5.45)	22 (5.63)
Previous department		
Internal medicine	88 (43.56)	32 (8.18)
Men’s section	0 (0)	1 (0.26)
Psychiatry	1 (0.5)	122 (31.2)
Emergency department	19 (9.41)	20 (5.12)
ICU	5 (2.48)	5 (1.28)
General branch	10 (4.95)	4 (1.02)
Imaging section	1 (0.5)	0 (0)
Laboratory section	0 (0)	1 (0.26)
Surgical department	37 (18.32)	15 (3.84)
Rehabilitation department	6 (2.97)	153 (39.13)
Logistics department	5 (2.48)	6 (1.53)
Gynecologic	6 (2.97)	3 (0.77)
Pediatric	9 (4.46)	9 (2.3)
Oncology	3 (1.49)	5 (1.28)
Infectious department	6 (2.97)	3 (0.77)
Chinese medicine	2 (0.99)	10 (2.56)
Five official sections	3 (1.49)	1 (0.26)
Dermatology	1 (0.5)	1 (0.26)
Level the hospital		
3A	158 (78.22)	267 (68.29)
3B	3 (1.49)	67 (17.14)
2A	15 (7.43)	51 (13.04)
2B	26 (12.87)	6 (1.53)
Current department		
ICU	9 (4.46)	7 (1.79)
Non-ICU	193 (95.54)	384 (98.21)
Change the working place		
No	156 (77.23)	348 (89)
Yes	46 (22.77)	43 (11)

### Health condition

Table 2 presents the health, work, and psychological status of the nurses in this study. Compared to nurses outside Hubei Province, those working in Wuhan were more likely to have a fever (3.5% vs. 0.8%), respiratory symptoms (18.8% vs. 1.3%), and systemic symptoms (11.4% vs. 1.0%) in the past 2 weeks (*p* < 0.0001). Consistently, the proportion of the grinding glass shadow via lung CT among those in Wuhan was higher than that outside Hubei (*p* = 0.002).

**Table 2:** Health condition of the nurses.

	Wuhan (%)/*N*	Non-Hubei (%)/*N*	*p*-value
Do you have a fever in the last 2 weeks			0.0363
No	195 (96.53)	388 (99.23)	
Yes	7 (3.47)	3 (0.77)	
Do you have respiratory symptoms in the last 2 weeks			<0.0001
No	164 (81.19)	386 (98.72)	
Yes	38 (18.81)	5 (1.28)	
Do you have systemic symptoms in the last 2 weeks			<0.0001
No	179 (88.61)	387 (98.98)	
Yes	23 (11.39)	4 (1.02)	
None			<0.0001
No	67 (33.17)	24 (6.14)	
Yes	135 (66.83)	367 (93.86)	
Sore throat			<0.0001
No	154 (76.24)	375 (95.91)	
Yes	48 (23.76)	16 (4.09)	
Anti-acid reflux			0.0213
No	196 (97.03)	389 (99.49)	
Yes	6 (2.97)	2 (0.51)	
Indigestion			<0.0001
No	185 (91.58)	387 (98.98)	
Yes	17 (8.42)	4 (1.02)	
Diarrhea			0.0004
No	189 (93.56)	387 (98.98)	
Yes	13 (6.44)	4 (1.02)	
Constipation			0.0002
No	182 (90.1)	382 (97.7)	
Yes	20 (9.9)	9 (2.3)	
Bloated			0.0006
No	192 (95.05)	389 (99.49)	
Yes	10 (4.95)	2 (0.51)	
Abdominal pain			0.1875
No	198 (98.02)	389 (99.49)	
Yes	4 (1.98)	2 (0.51)	
Other			0.015
No	192 (95.05)	385 (98.47)	
Yes	10 (4.95)	6 (1.53)	
The last 2 weeks, your lungs CT with out of the hint “lung grinding glass shadow”			0.0015
No	196 (97.03)	391 (100)	
Yes	6 (2.97)	.(.)	
The last month there is a shift?			<0.0001
No	44 (21.78)	151 (38.62)	
Yes	158 (78.22)	240 (61.38)	
On average, a few white shifts a week			0.7223
0–2	70 (34.65)	145 (37.08)	
3–5	99 (49.01)	178 (45.52)	
>5	33 (16.34)	68 (17.39)	
On average, several night shifts a week			<0.0001
0–2	129 (63.86)	329 (84.14)	
3–5	68 (33.66)	52 (13.3)	
>5	5 (2.48)	10 (2.56)	
The average length of each shift work is			<0.0001
<8 h	142 (70.3)	157 (40.15)	
8–16 h	59 (29.21)	228 (58.31)	
17–24 h	1 (0.5)	6 (1.53)	
Compared to the outbreak before, about the last 2 weeks of your work intensity, you think			<0.0001
It’s not very different	45 (22.28)	167 (42.71)	
It’s harder than before	92 (45.54)	169 (43.22)	
significantly harder than before	65 (32.18)	55 (14.07)	
Have you taken the following isolation measures?			<0.0001
No	39 (19.31)	271 (69.31)	
Self-isolation at home	22 (10.89)	68 (17.39)	
Separation from the family	141 (69.8)	52 (13.3)	
Are there any family members in your home who need to be cared for?			0.0002
No	80 (39.6)	196 (50.13)	
Old man	42 (20.79)	40 (10.23)	
Infants or children	66 (32.67)	143 (36.57)	
Pregnant women	.(.)	2 (0.51)	
People with disabilities	1 (0.5)	.(.)	
Other needs to be taken care of	13 (6.44)	10 (2.56)	
In the last 2 weeks, have your family had respiratory symptoms?			0.0002
No	187 (92.57)	385 (98.47)	
Yes	15 (7.43)	6 (1.53)	
In the last 2 weeks, do you have a family line CT check tips “lung grinding glass shadow”?			0.1157
No	200 (99.01)	391 (100)	
Yes	2 (0.99)	.(.)	
According to your feelings and experience, how often does the following situation appear to you? Work makes me feel physically and mentally exhausted			<0.0001
Never	12 (5.94)	95 (24.3)	
Occasionally	89 (44.06)	226 (57.8)	
Regularly	52 (25.74)	49 (12.53)	
Frequently	16 (7.92)	13 (3.32)	
Daily	33 (16.34)	8 (2.05)	
I feel exhausted after work			<0.0001
Never	12 (5.94)	89 (22.76)	
Occasionally	82 (40.59)	209 (53.45)	
Regularly	54 (26.73)	68 (17.39)	
Frequently	19 (9.41)	15 (3.84)	
Daily	35 (17.33)	10 (2.56)	
I feel very tired when I wake up in the morning and have to face a day of work			<0.0001
Never	30 (14.85)	165 (42.2)	
Occasionally	78 (38.61)	179 (45.78)	
Regularly	52 (25.74)	28 (7.16)	
Frequently	11 (5.45)	12 (3.07)	
Daily	31 (15.35)	7 (1.79)	
I doubt the significance of the work I do			<0.0001
Never	93 (46.04)	284 (72.63)	
Occasionally	71 (35.15)	82 (20.97)	
Regularly	19 (9.41)	17 (4.35)	
Frequently	5 (2.48)	4 (1.02)	
Daily	14 (6.93)	4 (1.02)	
According to your feelings and experience, how often does the following situation appear to you? I can effectively solve problems at work			0.0424
Never	2 (0.99)	13 (3.32)	
Occasionally	19 (9.41)	27 (6.91)	
Regularly	94 (46.53)	144 (36.83)	
Frequently	25 (12.38)	54 (13.81)	
Daily	62 (30.69)	153 (39.13)	
I feel I am making a contribution to the hospital			0.0252
Never	3 (1.49)	10 (2.56)	
Occasionally	28 (13.86)	27 (6.91)	
Regularly	73 (36.14)	129 (32.99)	
Frequently	20 (9.9)	34 (8.7)	
Daily	78 (38.61)	191 (48.85)	
In my opinion, I am good at my job			0.0034
Never	2 (0.99)	13 (3.32)	
Occasionally	25 (12.38)	21 (5.37)	
Regularly	79 (39.11)	137 (35.04)	
Frequently	28 (13.86)	47 (12.02)	
Daily	68 (33.66)	173 (44.25)	
I am confident that I can do all the work effectively			0.0088
Never	2 (0.99)	10 (2.56)	
Occasionally	17 (8.42)	15 (3.84)	
Regularly	79 (39.11)	127 (32.48)	
Frequently	29 (14.36)	46 (11.76)	
Daily	75 (37.13)	193 (49.36)	

### Working conditions

As shown in Table 2, participants in Wuhan had more frequent work shifts, especially night shifts (*p* < 0.0001). Compared with their regular work schedule before the coronavirus disease outbreak, the COVID-19-related work was significantly harder in terms of work intensity in the last 2 weeks, which was more prominent in the Wuhan group (*p* < 0.0001). Besides, the participants in Wuhan were likely to feel physically and mentally exhausted, compared with the colleagues outside Hubei province (*p* < 0.0001).

### Psychological responses

#### Probable depression and depressive symptoms

As shown in Table 3, the PHQ-9 scores in each item and in a total of the Wuhan group were significantly higher than their counterparts (*p* < 0.0001). Probable depression was reported by 31.2% of participants in Wuhan, which was significantly higher than those outside Hubei province (13.8%). Correspondingly, depressive symptoms were more often reported in the Wuhan group (70.8%) compared with those outside Hubei (41.4%) (*p* < 0.0001).

**Table 3:** Outcomes of PHQ-9 for nurses.

Items	Wuhan (*N* = 202) Mean ± SE	Non-Hubei (*N* = 391) Mean ± SE	*p-*value
1. Little interest or pleasure in doing things	1.01 ± 0.06	0.64 ± 0.04	<0.0001
2. Feeling down, depressed, or hopeless	0.91 ± 0.06	0.48 ± 0.03	<0.0001
3. Trouble falling or staying asleep, or sleeping too much	1.35 ± 0.07	0.76 ± 0.04	<0.0001
4. Feeling tired or having little energy	1.23 ± 0.07	0.71 ± 0.04	<0.0001
5. Poor appetite or overeating	1.1 ± 0.06	0.66 ± 0.04	<0.0001
6. Feeling bad about yourself or that you are a failure or have let yourself or your family down	0.75 ± 0.06	0.43 ± 0.04	<0.0001
7. Trouble concentrating on things, such as reading the newspaper or watching television	0.88 ± 0.06	0.46 ± 0.04	<0.0001
8. Moving or speaking so slowly that others could have noticed, or being so fidgety/restless that you have been moving more than usual	0.73 ± 0.06	0.36 ± 0.03	<0.0001
9. Thoughts you would be better off dead, or of hurting yourself	0.49 ± 0.06	0.22 ± 0.03	<0.0001
Total	8.44 ± 0.47	4.71 ± 0.26	<0.0001

	*N*	Percent (%)	*N*	Percent (%)
Total PHQ ≥10	63	31.19	54	13.81
Total PHQ 0–4	59	29.21	229	58.57
Total PHQ 5–9	80	39.6	108	27.62
Total PHQ 10–14	33	16.34	29	7.42
Total PHQ 15–19	15	7.43	20	5.12
Total PHQ 20–27	15	7.43	5	1.28

#### Anxiety symptoms

Similar to the PHQ-9 scores, the GAD-7 scores of the Wuhan group were significantly higher than those of the non-Hubei group (*p* < 0.0001). Probable anxiety was reported by 18.3% of participants in Wuhan, which was significantly higher than the counterparts (5.9%), as presented in Table 4.

**Table 4:** Outcomes of GAD-7 for nurses.

Items	Wuhan (*N* = 202)	Non-Hubei (*N* = 391)	*p*-value
	Mean ± SE	Mean ± SE	
1. Feeling nervous, anxious, or on edge	1.05 ± 0.06	0.56 ± 0.03	<0.0001
2. Not being able to stop or control worrying	0.91 ± 0.06	0.42 ± 0.03	<0.0001
3. Worrying too much about different things	0.86 ± 0.06	0.43 ± 0.03	<0.0001
4. Trouble relaxing	0.79 ± 0.06	0.41 ± 0.03	<0.0001
5. Being so restless that it’s hard to sit still	0.68 ± 0.06	0.29 ± 0.03	<0.0001
6. Becoming easily annoyed or irritable	0.84 ± 0.06	0.46 ± 0.03	<0.0001
7. Feeling afraid as if something awful might happen	0.74 ± 0.06	0.35 ± 0.03	<0.0001
Total score	5.86 ± 0.40	2.91 ± 0.20	<0.0001

	*N*	Percent (%)	*N*	Percent (%)
Total score ≥10	37	18.32	23	5.88
Total score 0–4	91	45.05	277	70.84
Total score 5–9	74	36.63	91	23.27
Total score 10–13	9	4.46	8	2.05
Total score 14–18	17	8.42	12	3.07
Total score 19–21	11	5.45	3	0.77

#### Seeking mental health services

As shown in Table 5, most of the nurses never received the professional psychological assistance in the Wuhan (96.0%) and non-Hubei (95.9%) group. In both groups, the rest participants mainly received assistance for free, from the counselor, online, and also from doctors outside Hubei province. Compared with the non-Hubei group, the Wuhan group was more likely to administer psycho-pharmaceutical drugs.

**Table 5:** Mental health service for nurses.

	Wuhan (*N*, %)	Non-Hubei (*N*, %)
Have you received professional psychological assistance?		
No	194 (96.04)	375 (95.91)
Yes	8 (3.96)	16 (4.09)
What kind of professional psychological assistance have you received?		
Skip	0	2 (0.51)
No	194 (96.04)	375 (95.91)
Paid service	1 (0.5)	3 (0.77)
Free service	7 (3.47)	11 (2.81)
What kind of expert assistance have you received?		
Skip	0	3 (0.77)
No	194 (96.04)	375 (95.91)
Psychiatrist	0	2 (0.51)
Counselor	6 (2.97)	8 (2.05)
Social worker	1 (0.5)	2 (0.51)
Other	1 (0.5)	1 (0.26)
What kind of psychological assistance has been received?		
Skip	0	3 (0.77)
No	194 (96.04)	375 (95.91)
On-site consultation	3 (1.49)	6 (1.53)
Online consultation	5 (2.48)	7 (1.79)
What sources of psychological assistance have you received?		
Skip	0	5 (1.28)
No	194 (96.04)	375 (95.91)
Doctors in Hubei province	3 (1.49)	1 (0.26)
Doctors outside Hubei province	5 (2.48)	10 (2.56)
Do you take a sedative or hypnotic drugs?		
No	176 (87.13)	374 (95.65)
Yes	26 (12.87)	17 (4.35)
Do you take antidepressant and anxiety drugs?		
No	195 (96.53)	387 (98.98)
Yes	7 (3.47)	4 (1.02)

#### Social support networks

Nurses in both Wuhan and non-Hubei groups received family support. Compared with the non-Hubei group, Table 6 shows that the Wuhan group received less social support according to the reported SSRS total scores (mean score: 39.6 vs. 42.35, *p* < 0.0001).

**Table 6:** Outcomes of SSRS for nurses.

	Wuhan		Non-Hubei		*p-*value
	Mean	SD	Mean	SD	*p*
Objective support score (2 + 6 + 7) (mean, std)	8.88	3.72	9.72	3.55	<0.0001
Subjective support score (1 + 3 + 4 + 5) (mean, std)	23.23	5.60	24.43	5.21	<0.0001
Support utilization (8 + 9 + 10) (mean, std)	7.50	2.10	8.19	2.01	<0.0001
Total score (mean, std)	39.60	9.57	42.35	8.68	<0.0001

## Discussion

To our knowledge, this is the first comparative study on psychological responses focused on depression and anxiety in frontline healthcare nurses. The main findings show that psychological status among nurses in Wuhan was more depressive and anxious with less social support and worse health condition when compared with their colleagues in other cities outside Hubei Province. In addition, we observed that only a small number of nurses sought professional psychological assistance.

The results revealed that characters of COVID-19-related psychological responses included a high level of depression. In our study, we adopted the PHQ-9 to screen the depressive state in the two nurse groups^12^ based on semi-structured interviews such as Structured Clinical Interview for DSM Disorders (SCID). High-level psychological pressure may lead to depression. In 2003, another large-scale infectious threat, severe acute respiratory syndrome (SARS), caused significant psychological stress. Since SARS has similar epidemic characteristics with the COVID-19, it is to be expected that today frontline healthcare workers throughout the world will show manifestations of high-level stress. In the work by Tam et al., the authors adopt the General Health Questionnaire to identify psychological distress. In that study, 68% of Hong Kong frontline healthcare workers showed high-level stress^13^. In our study, 31.2% of the nurses of the Wuhan group were reported with a high-level depression and presented more severe psychological symptoms, which were significantly higher than those of nurses outside Hubei province (13.8%).

Moreover, we found that healthcare nurses in the Wuhan group had significantly higher anxiety than the Non-Hubei group, according to the GAD-7^14^. Chen’s research determined that SARS nursing staff members were anxious and depressed in Taiwan^15^. Both SARS and the 2019-nCoV required health workers to be equipped with full-body protective gear. Based on our data, 48.4% of nurses worked continuously for 8–16 h shifts during this period of the outbreak. Probable anxiety was reported by 18.3% of nurses in the Wuhan group, which was significantly higher than that of their counterparts (5.9%). As of February 8, 2020, ~30 medical staff in Wuhan Mental Health Center (WMHC) were diagnosed with 2019-nCoV^16^. The nursing staff could not eat, drink, or use the bathroom. The nursing staff was potentially easily infected when they performed routine work, such as sputum suction. Under these trying conditions, nursing staff understandably become physically and mentally burned out, leading to anxiety and depression^15^. All the above factors would significantly increase the anxiety of frontline medical workers. In 2014, the Middle East Respiratory Syndrome Corona Virus (MERS-CoV) breakout was associated with a high incidence of anxiety in medical students studying in teaching hospitals in affected countries. 100% of the students reported anxiety, most of them reported minimal anxiety, and 4.6% reported moderate anxiety^17^.

During the lunar new year holiday travel peak, massive numbers of people, including some infected with COVID-19, traveled from Wuhan to every province in China as far as Tibet. Another five million people fled Wuhan the day prior to the Wuhan Lockdown. By March 4, 2020, there were 80,422 confirmed cases of COVID-19 in China, and 83.7% of the cases (67,332) were from Hubei Province. Although the number of cases outside the Hubei province was relatively low, the infection rate was similarly high^18^. The population all over China, including nursing staff, would be highly alert of the disease. On the other hand, nurses in some departments related to COVID-19 triage, diagnosis, and the treatment worked harder than before. Finally, because of the COVID-19 episode, some of the nursing staff have had to be quarantined from their family. The vast majority of nursing staff are young women without siblings because of the one-child policy with one or two children and elderly parents to care for. Such demanding circumstances could contribute further to their depression and anxiety^19^.

There was an interplay between the physical health condition and mental health^20^. In China, Wuhan was the outbreak area of the COVID-19 in which the most severe periods were February and March 2020. The healthcare nurse in Wuhan had higher depression and anxiety levels than that outside of Wuhan because the medical supplies were in short and working hours had been highly increased in Wuhan. In the case of close contact and care of patients, healthcare nurses in Wuhan were under great psychological pressures with overloaded physical bodies and nerves. These had led to a decline in their immunity and body fatigue. Therefore, when compared with healthcare nurses outside Wuhan, they had a worse health condition, which in turn might affect their psychological responses by aggravating their depressive and anxious emotions.

The results of the Wuhan group were worse than those of the non-Hubei group in the three dimensions of social support, and their worse results in objective support, subjective support, and support utilization might lead to depressive and anxious emotions. So far, psychiatrists and psychologists all over China have contributed actively to helping the public and nursing staff to better respond to the stress-related to the COVID-19^4^ pandemic. However, our data showed nursing staff received social-psychological support and psychological comfort mainly from family members, and only about 4% of the nurses received professional assistance from a mental health care service. Since the quarantine of diagnosed cases and limiting inter-personal contact were efficient methods for controlling the spread of COVID-19^21^, these approaches have been widely adopted in China, especially in Wuhan. Therefore, quarantine makes face-to-face psychological counseling difficult. Nowadays, however, internet services and smartphones enable psychological workers to provide online services during the COVID-19 outbreak. Currently, the WeChat-based Survey Program Questionnaire Star that targets medical staff could be used to collect important psychological information for the mental health workers. Free online mental education, as well as online psychological counseling services, has also been established widely^22^. Structured letter therapy, which is a kind of remote written counseling, has become a new psychological counseling mode^23^. On February 21, 2020, Shanghai deployed the 9th group medical staff, which has 50 psychologists and psychiatrists, to Wuhan. The Shanghai mental health center has also sent psychiatrists regularly to Shanghai local COVID-19 special hospitals. Although the methods mentioned above were considerable and efficient, compared with searching for psychological assistance, the nursing staff preferred to have a rest or even sleep to release the fatigue and pressure after the overloaded work^4^. Therefore, they did not have the time and energy to receive psychological assistance. It might continue to reduce the social support they received and led to increased depression and anxiety levels.

During the outbreak, nurses around the world may also face the same psychological impacts that reduce their work efficiency. The current epidemic situation in China has a positive tendency, while, as of April 8, 2020, the world is still in the outbreak period. In many countries, there is a shortage of medical supplies, and many medical workers could not receive medical protection under the incredible work pressure, so they also suffered substantial psychological trauma. In the outbreak of COVID-19, an American medical worker posted a video that attracted hundreds of millions of views. Being crying to the camera with the complaint of the lack of medical protection equipment in the United States, she had to choose to quit to protect herself. It also may lead to the psychological panic of medical staff around the world.

The above reflects the need for optimizing the construction of the nurse emergency psychological assistance system during the epidemic outbreak. At the center of epidemics, medical workers will inevitably be under tremendous pressure, and the primary purpose of psychological workers is to help them relieve mental stress and improve work efficiency. However, although we have invested a lot of psychological and material resources^20^, nurses in Wuhan were more depressive and anxious with less social support. It indicates that reserve resources, coverage, management system, and the effectiveness of the current psychological assistance for fighting with COVID-19 are not satisfactory, and all of them need to be optimized. Therefore, the psychological resources should be integrated, thus making contributions to the construction and optimization of the nurse emergency psychological assistance system in epidemics.

This study has several limitations. First, psychological status, including depression and anxiety, were all rated subjectively by using scores and therefore, might be inaccurate. Second, as participants were included in our study on a voluntary basis, there may be a response bias amongst the volunteers. Third, selection bias may exist because the participants were only from a limited number of hospitals in Wuhan and other cities. Fourth, this study cannot escape the limitation of cross-sectional studies. We cannot explore the continuity of changes in the psychological responses of healthcare nurses in time series.

In summary, our study demonstrates that the prevalence of psychological pressure is high among frontline nurses, especially those in the center of epidemics, such as Wuhan and that it contributes to significant mental health issues such as anxiety and depression and influences their work efficiency. Appropriate psychological intervention should be provided early in such a crisis to support staff, to maintain their mental health and work functionality during such stressful circumstances. These enlighten authorities and researchers to pay more attention to the reform of the nurse emergency psychological assistance system, psychological assistance talent training, psychological assistance knowledge popularization to deal with unpredictable epidemic outbreaks in the future.

## References

[1] Guan, W. J. et al. Clinical characteristics of 2019 novel coronavirus infection in China. *N. Engl. J. Med.*10.1101/2020.02.06.20020974 (2020).

[2] Nkengasong JN, Mankoula W (2020). Looming threat of COVID-19 infection in Africa: act collectively, and fast. Lancet.

[3] Moss P, Barlow G, Easom N, Lillie P, Samson A (2020). Lessons for managing high-consequence infections from first COVID-19 cases in the UK. Lancet.

[4] Chen Q (2020). Mental health care for medical staff in China during the COVID-19 outbreak. Lancet Psychiatry.

[5] Huang, Y. & Zhao, N. Mental health burden for the public affected by the COVID-19 outbreak in China: Who will be the high-risk group? *Psychol. Heal. Med*. 1–12 (2020). 10.1080/13548506.2020.1754438 32286091

[6] Sun K, Chen J, Viboud C (2020). Early epidemiological analysis of the coronavirus disease 2019 outbreak based on crowdsourced data: a population-level observational study. Lancet Digit. Heal..

[7] Wu, Z. & McGoogan, J. M. Characteristics of and important lessons from the coronavirus disease 2019 (COVID-19) outbreak in China: Summary of a report of 72 314 cases from the Chinese Center for Disease Control and Prevention. *JAMA* **323**, 1239–1242 (2020). 10.1001/jama.2020.2648 32091533

[8] Chen S (2020). Fangcang shelter hospitals: a novel concept for responding to public health emergencies. Lancet.

[9] Kroenke K, Spitzer RL, Williams JB (2001). The PHQ-9: validity of a brief depression severity measure. J. Gen. Intern. Med..

[10] Spitzer RL, Kroenke K, Williams JBW, Löwe B (2006). A brief measure for assessing generalized anxiety disorder: the GAD-7. Arch. Intern. Med..

[11] Ke X, Liu C, Li N (2010). Social support and Quality of Life: a cross-sectional study on survivors eight months after the 2008 Wenchuan earthquake. BMC Public Health.

[12] Levis B, Benedetti A, Thombs BD (2019). Accuracy of Patient Health Questionnaire-9 (PHQ-9) for screening to detect major depression: individual participant data meta-analysis. BMJ.

[13] Tam CWC, Pang EPF, Lam LCW, Chiu HFK (2004). Severe acute respiratory syndrome (SARS) in Hong Kong in 2003: stress and psychological impact among frontline healthcare workers. Psychol. Med..

[14] Hinz A (2017). Psychometric evaluation of the Generalized Anxiety Disorder Screener GAD-7, based on a large German general population sample. J. Affect. Disord..

[15] Chen R (2006). Effects of a SARS prevention programme in Taiwan on nursing staff’s anxiety, depression and sleep quality: a longitudinal survey. Int. J. Nurs. Stud..

[16] Zhu Y (2020). The risk and prevention of novel coronavirus pneumonia infections among inpatients in psychiatric hospitals. Neurosci. Bull..

[17] Al-Rabiaah A (2020). Middle East Respiratory Syndrome-Corona Virus (MERS-CoV) associated stress among medical students at a university teaching hospital in Saudi Arabia. J. Infect. Public Health.

[18] Chen, Z. et al. Distribution of the 2019-nCoV epidemic and correlation with population emigration from Wuhan, China. *medRxiv*10.1101/2020.02.10.20021824 (2020). 10.1097/CM9.0000000000000782 PMC7147281 32118644

[19] Brooks SK (2020). The psychological impact of quarantine and how to reduce it: rapid review of the evidence. Lancet.

[20] Pinquart M, Sörensen S (2003). Differences between caregivers and noncaregivers in psychological health and physical health: a meta-analysis. Psychol. Aging.

[21] Hellewell J (2020). Feasibility of controlling COVID-19 outbreaks by isolation of cases and contacts. Lancet Glob. Health.

[22] Liu S (2020). Online mental health services in China during the COVID-19 outbreak. Lancet Psychiatry.

[23] Xiao C (2020). A novel approach of consultation on 2019 novel coronavirus (COVID-19)-related psychological and mental problems: structured letter therapy. Psychiatry Investig..

